# Is Endotoxemia in Stable Hemodialysis Patients an Artefact? Limitations of the Limulus Amebocyte Lysate Assay and Role of (1→3)-β-D Glucan

**DOI:** 10.1371/journal.pone.0164978

**Published:** 2016-10-20

**Authors:** Jonathan Wong, Yonglong Zhang, Ashish Patidar, Enric Vilar, Malcolm Finkelman, Ken Farrington

**Affiliations:** 1 Lister Renal Unit, Hertfordshire, United Kingdom; 2 University of Hertfordshire, Hertfordshire, United Kingdom; 3 Associates of Cape Cod Inc., East Falmouth, Massachusetts, United States of America; Postgraduate Medical Institute, INDIA

## Abstract

**Background:**

Elevated blood endotoxin levels are frequently reported in the dialysis population and are strongly linked with inflammation, a major predictor of mortality. Virtually all studies have employed the Limulus Amoebocyte Lysate (LAL) assay to detect endotoxin. However this assay is not endotoxin-specific and can be activated by (1→3)-β-glucan (BG), a component of fungal cell walls leading to false positive signals. Very few studies have taken account of this. We examined the influence of BG-based activation of the LAL assay on the detection of endotoxemia in this setting.

**Method:**

We measured plasma endotoxin levels in 50 hemodialysis patients with and without the use of BG-blocking buffers. These buffers inhibit BG activation of the LAL assay to ensure that any signal detected is endotoxin-specific. Blood samples were measured for BG, interleukin-6 (IL-6), tumor necrosis factor-alfa (TNF-α) to examine the association between endotoxin signals, BG and inflammation.

**Results:**

Endotoxin signals were detected in 50% of patients. On repeat measurement with a BG-blocking buffer, all detected endotoxin signals were extinguished. No patient had detectable endotoxemia. Plasma BG levels were significantly elevated in 58% of patients and were higher in those with detectable endotoxin signals using the LAL assay without BG-blocking buffers (78vs.54pg/mL;p<0.001). Endotoxin signal and BG levels did not correlate with levels of TNF-α or IL-6.

**Conclusion:**

Use of the LAL assay for blood endotoxin detection in dialysis patients has its limitations due to high blood BG. Endotoxemia frequently reported in non-infected hemodialysis patients may be artefactual due to BG interference.

## Introduction

Endotoxemia is commonly reported in the dialysis population and has been associated with systemic inflammation[1–7]–a strong prognostic of poor outcome[8]. Endotoxins are complex lipopolysaccharides found in the outer cell wall of gram negative bacteria. They are implicated in the pathogenesis of sepsis syndrome and are potent mediators of inflammation. The levels of endotoxemia reported in the dialysis population range from 0.209 to 2.31 endotoxin units/mL (EU/mL)[2], [3], [5], [7], [9–12]. This appears high since it is well established that 4.1–5 EU/kg/hr is sufficient to induce pyrogenic symptoms such as rigor, nausea and hypotension in humans [13], [14]. Assuming an average 70kg patient with approximately 3L circulating plasma volume[15], it would be expected that as little as 0.12 EU/mL would be sufficient to trigger pyrogenic symptoms. Reported endotoxemia in the dialysis population exceeds this threshold.

Nearly all studies in the dialysis population have employed the use of the LAL assay to detect endotoxin[9]. However, the LAL assay has many limitations especially when used to measure endotoxin in complex biological substances such as plasma. LAL is a very sensitive biological assay, capable of detecting sub-picogram/mL levels of endotoxin. The assay was originally thought to be specific to endotoxin though it is now understood that it can also be activated by (1→3)-β-D-glucan (BG) via an alternate enzymatic pathway mediated by factor G activation[16]. BGs are major carbohydrate constituents of cereal, yeast and fungal cell walls[17] with a variable molecular weight ranging from thousands to millions of daltons depending on origin[17], [18]. The presence of elevated BG in the blood has been used as a surrogate marker of invasive fungal infection[17]. False positive activation of LAL due to BG interference may be partly responsible for the elevated blood endotoxin levels frequently reported in the literature. Taniguchi et al [19] found that endotoxin levels were much lower in hemodialysis patients when blood samples were assayed using LAL devoid of factor G compared with standard unmodified LAL, although they did not measure BG levels in their study.

It is important to clarify whether blood endotoxin levels are truly raised in dialysis patients since emerging therapies such as extracorporeal endotoxin are in development and their efficacy in the treatment of sepsis syndrome has been reported [20]. These therapies could potentially be applied to hemodialysis patients for treatment of chronic inflammation—a potentially major benefit since targeted anti-inflammatory interventions are currently unavailable.

LAL assay reactivity to BG can be prevented by either using LAL reagent that lacks factor G or by rendering the factor G component of the LAL assay unreactive to BG using BG-blocking buffers. In a recent review of endotoxin studies in dialysis population[9], with one notable exception[19], studies did not specify the use of LAL rendered insensitive to BG activation. Hence it is unclear whether reported high levels of endotoxemia are truly due to endotoxin or due to false positive interference from BG.

We recently examined the performance of a kinetic turbidimetric LAL assay in HD patients and found that the assay was sensitive and precise for endotoxin detection in uraemic plasma[21], [22]. Using this assay, we investigated the influence of BG on endotoxin read-out measurements by measuring endotoxin in HD patients with and without the use of a BG-blocking buffer. In a separate experiment, in order to determine whether use of BG-blocking buffers interfered with the detection of true endotoxemia, we spiked plasma samples with known amounts of endotoxin, and measured endotoxin levels with and without the blocking buffer. BG was measured in plasma samples to determine its association with endotoxin.

## Method

### Study design

This was a single centre cross-sectional study of prevalent HD patients treated at the Lister Renal Unit. Ethical approval was obtained from the Northampton NHS Research Ethics Committee and the study was conducted in accordance with the Declaration of Helsinki. Written informed consent was obtained from all participants. All patients dialysed using an arterio-venous fistula and were clinically stable at the time of study. All were treated by on-line hemodiafiltration using ultrapure water. Exclusion criteria were HIV infection, viral hepatitis, abnormal liver function and active gastrointestinal disease.

The study was divided into two phases. The first phase compared the difference in endotoxin read-out measurements between standard LAL (without BG-blocking buffer **[LAL(-)])** and LAL reconstituted with manufacturer supplied BG-blocking buffer (Charles Rivers^®^ ES-buffer [**LAL(+)**]) to render the LAL insensitive to further stimulation by BG. The second phase of the study was carried out to ensure that BG-blocking buffers themselves do not affect the accuracy or interfere with the sensitivity of LAL to detect endotoxin.

### Phase 1: Comparison of endotoxin measurements between LAL(-) and LAL(+)

Fifty patients were recruited. For each subject, plasma samples were assayed for endotoxin using both LAL(-) and LAL(+). Samples were also measured for IL-6, TNF-α and BG. Correlation between BG, markers of inflammation, demographic and clinical factors was explored.

### Blood sampling and processing

Blood samples were collected pre-dialysis through the arterio-venous fistula using aseptic technique. Blood samples for endotoxin and BG measurements were collected in Terumo Venoject II heparinised tubes (Project KBG, Tokyo). Blood samples for cytokine measurements were collected in S-monovette Z-gel tubes (Sarsedt, Germany). Plasma for endotoxin and BG measurements were prepared by centrifugation at 250g for 10min and serum for cytokine measurements were prepared by centrifugation at 1500g for 10min. Plasma and serum samples were immediately frozen and stored at -80°C. Blood collection, processing and storage were completed within 30 minutes for all samples. Phlebotomy equipment including syringes and blood collection tubes were batch checked for endotoxin contamination[21], [23]. Washout from the apparatus were consistently found to have undetectable endotoxin (<0.0025 EU/mL).

### Laboratory measurements

#### Endotoxin assay using standard LAL without BG-blocking buffer [LAL(-)]

Endotoxin measurements were performed using the kinetic turbidimetric LAL assay (Endosafe KTA2, Charles River Laboratories, Ecully) as previously described[21]. Plasma samples were diluted 1:10 with 0.1% Tween80 (Merck Chemicals, Darmstadt) and heated to 70°C for 10min and cooled to room temperature (20–25°C) prior to analysis. 100μL of diluted plasma was added to duplicate wells on 96-well microplates. Endosafe KTA2 reagent was reconstituted with 5.2mL LAL reagent water. 100μL of this mixture was added to each sample. The plate was monitored at 340nm using a Biotek ELx808 absorbance microplate reader with Endoscan-V software (version 4.0; Charles River) with an onset optical density set at 0.03. Six-point standard curves were constructed using standard dilutions of control standard endotoxin (*E*.*coli* 055:B5) ranging from 10–0.0025 EU/mL. All standard curves had a correlation coefficient >0.98. Coefficient of variation (CV) for onset reaction times for each assay was <20%.

#### Endotoxin assay using LAL reconstituted with BG-blocking buffer [LAL(+)]

To block the factor G pathway, Endosafe KTA2 reagent was reconstituted with 5.2mL BG-blocking buffer (Charles River ES-Buffer) containing 1mg/mL carboxymethylated curdlan[24], [25]. Endotoxin measurements were carried out using the same procedure as described above. For each patient, samples were analysed with LAL(-) and LAL(+) simultaneously on the same microplate. The same batch of LAL reagent was used throughout the study.

#### (1–3)-β-D glucan assay

BG measurements was carried out using the Fungitell^®^ assay (Associates of Cape Cod, Inc.) as per manufacturer’s instructions[26], [27]. In brief, 5μL of sample was mixed with 20μL pre-treatment buffer (0.125M KOH/6M KCl), in microplate wells, and incubated at 37°C for 10min. Fungitell reagent, reconstituted in 0.1M Tris HCl, pH 7.4, was added to sample and standard curve wells (7.8–500pg/mL, Pachyman). The reactions were read kinetically, at 405nm minus 490nm at 37°C, for 40min. Vmean values (milliabsorbance units/min) were calculated for standards and samples and sample titres interpolated from the standard curve. CV for all assays was <20%. Normal human serum contains low levels of BG, typically 10-40pg/mL[28]. Levels <60pg/mL are interpreted as negative and between 60-80pg/mL is interpreted as indeterminate. Levels >80pg/mL is interpreted as positive and in at-risk patients is considered a marker of invasive fungal infection[26], [29].

#### Cytokine measurements and C-reactive protein measurements

serum was measured for IL-6 and TNF-α using enzyme-linked immunosorbent assays (Human Quantikine ELISA, R&D systems). For IL-6, intra- and inter-assay CV was 1.6–4.2% and 2.0–3.7% respectively. For TNF-α, intra- and inter-assay CV was 4.2–5.2% and 4.6–7.4% respectively. All patients at our unit have monthly CRP measurements (Olympus AU2700, Beckman-Coulter) as part of routine clinical care. Monthly CRP measurements for all patients were collected in the preceding 3 months of the study to determine the chronic inflammatory status of subjects.

#### Measurement of BG in dialysis fluid

Dialyzers were investigated for BG contamination. Dialysis priming fluid from 12 randomly selected dialyzers (10 Fresenius FX and 2 Gambro Evodial dialyzers) was measured for BG as described above. The limit of detection of BG in aqueous solutions is 7.8pg/mL.

### Phase 2: Investigating the effect of BG-blocking buffers on endotoxin spike recovery

**Fig 1:**
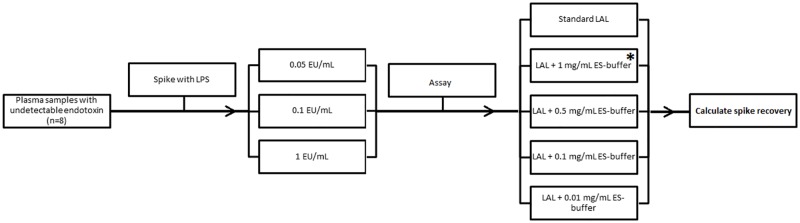
Schematic presentation of spiking experiments—plasma from each patient was aliquoted and spiked with three different concentrations of control standard endotoxin (LPS) and each spiked sample was assayed using LAL reconstituted with different doses of glucan blocking buffer to calculate % spike recovery. * denotes manufacturer recommended dose of glucan blocking buffer (Charles Rivers ES-buffer ^®^; Endotoxin-Specific buffer) containing 1 mg/mL of carboxymethylated curdlan.

To ensure that BG-blocking buffers themselves do not interfere with endotoxin detection, the ability of LAL reconstituted with different doses of BG-blocking buffers to detect endotoxin from plasma samples spiked with control standard endotoxin (lipopolysaccharide; LPS) was explored (Fig 1).

Eight patients with undetectable endotoxemia were recruited for this sub-study. Plasma from each subject was aliquoted and spiked with three different concentrations of LPS (0.05, 0.1 and 1EU/mL). Each spiked sample was diluted, heat-treated as described above and assayed with LAL(-) and LAL reconstituted with different concentrations of BG-blocking buffer. The standard inhibitor concentration of Charles River ES-buffer used to render LAL unreactive to BG stimulation is 1mg/mL carboxymethylated curdlan[24]. Each LPS-spiked plasma sample was assayed with LAL reconstituted with progressively diluted concentrations of carboxymethylated curdlan ranging from 0.01-1mg/mL to calculate percentage spike recovery. Spike recovery was calculated using the following formula: -

$$\%\;spike\;recovery=\frac{Measured\;endotoxin\;content\;in\;sample}{Amount\;of\;endotoxin\;added\;to\;sample}\times \;100\%$$

Spike recoveries between 50–200% are considered acceptable. The wide acceptable range is due to the fact that the LAL test is a biological assay and thus inherent variability in LAL testing is a well-recognised phenomenon due to differences in assay sensitivity, manufacturer reagents and laboratory accessories [30]. The sensitivity of each lot of commercial LAL formulations (originally gel clot formulations) is calibrated against Reference Standard Endotoxin (RSE) supplied by the Food and Drug Administration (FDA) or the United States Pharmacopoeia (USP). The concentration of endotoxin at which clot formation occurs is termed lambda. The USP states that the verification of a LAL test’s proper performance (i.e. lack of interference by the sample) is demonstrated by clot formation at ½ lambda, lambda, or twice lambda. This requirement gave rise to the 50–200% recovery specification used to verify appropriate LAL test performance[30], [31].

### Statistical analysis

Data are presented as means and medians with respective 95% confidence intervals and interquartile ranges. Paired and non-paired data were compared using Wilcoxon-signed rank and Mann-Whitney U test respectively. Ordinal data between multiple groups was compared using Kruskal-Wallis test. Correlation was compared using Spearman’s correlation coefficient. ROC analysis was used to identify optimum cut-off levels of BG to identify patients with detectable endotoxemia using the LAL(-). Level of agreement between patients with detectable endotoxemia and those with elevated BG levels was analysed using the kappa statistic.

## Results

### Patient characteristics

Patient characteristics are listed in Table 1. Mean age was 64 and median dialysis vintage was 2.8years. With the exception of one patient who dialyzed using synthetic polycarbonate heparin-grafted dialyzer (Gambro, Evodial), all were treated with synthetic polysulfone dialyzer (Fresenius, FX high-flux). Most had evidence of inflammation, with elevated IL-6 and TNF-α. 58% of patients had evidence of chronic inflammation with median CRP >5mg/L in the three months preceding the study.

**Table 1:** Patient characteristics.

Parameter	Value
Age (years)	64 [95% CI, 60–69]
Male gender (%)	74
Weight (kg)	78.2 [95% CI, 73.1–83.3]
CCI	3.8 [95% CI, 3.1–4.5] 4 [IQR 2–5]
Diabetes (%)	30
Previous gastrointestinal disease (%)	14
KRU (ml/min)	0.1 [IQR 0–2.0]
Kt/V	1.44 [95% CI, 1.36–1.51]
Dialysis vintage (years)	2.8 [IQR 1.8–7.8]
Convective volume (L)	18.8 [95% CI, 16.6–23.0]
CRP (mg/L)	7.0 [IQR 5–11.3]
High CRP ≥ 5 mg/L (%)	58%
Albumin (g/L)	38.4 [95% CI, 37.4–39.4]
TNF-α (pg/mL)	13.6 [IQR 11.6–17.7]
IL-6 (pg/mL)	7.7 [IQR 4.6–14.8]
PTH (pmol/L)	38.6 [IQR 29.0–54.5]
Dialyzer surface area (m^2^)	1.8 [IQR 1.8–2.2]

CCI, Charlson co-morbidity index; KRU, residual urea clearance; CRP, median C-reactive protein over the previous 3 months; PTH, parathyroid hormone; TNF-α, tumour necrosis factor-α; IL-6, interleukin-6 (values quoted for healthy controls for IL-6 and TNF-α are <3.1pg/mL and <2.8pg/mL respectively); Kt/V, combined dialyser and renal urea clearance normalised to body volume

### Effect of BG-blocking buffers on plasma endotoxin detection

Using LAL(-), 50% of patients had detectable endotoxemia with median endotoxin level of 0.038EU/mL, however on repeat measurement with LAL(+), no subjects had detectable endotoxemia (Table 2).

**Table 2:** Plasma endotoxin and (1–3)-β-D-glucan levels in patients with and without endotoxemia.

Population	Plasma endotoxin (EU/mL)		Plasma BG (pg/mL)
	LAL(-) (No BG blocker)	LAL(+) (with BG blocker)	
Detectable endotoxemia (n = 25)	0.038 [IQR 0.031–0.043]	Undetectable (<0.025)	78 [IQR 59–117]
No endotoxemia (n = 25)	Undetectable (<0.025)	Undetectable (<0.025)	54 [IQR 36–65]
p-value			<0.001

### Association between endotoxin signals detected using LAL(-) and (1–3)-β-D-glucan

**Fig 2:**
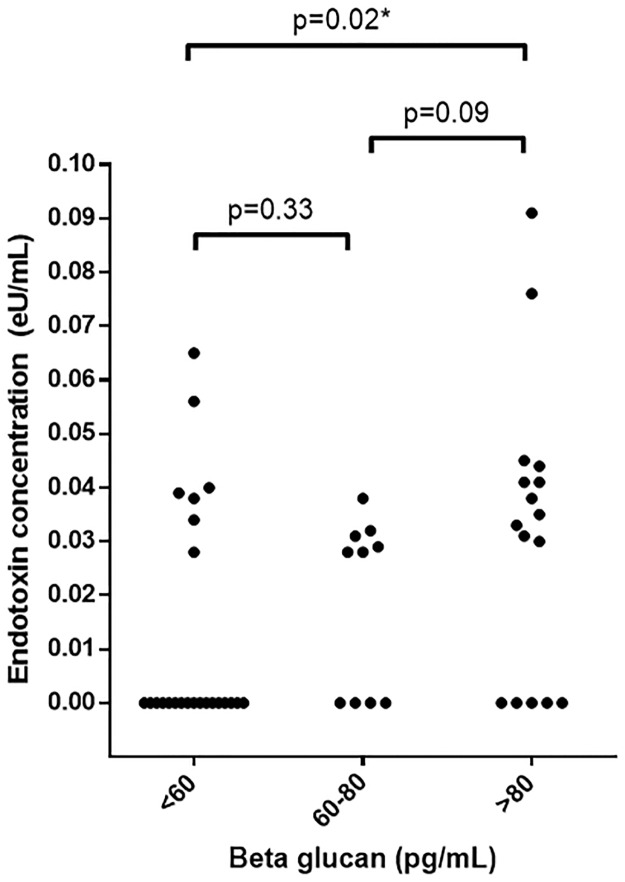
Endotoxin signal detected using standard LAL without BG blocking buffer displayed by tertiles of (1–3)-β-D glucan.

**Fig 3:**
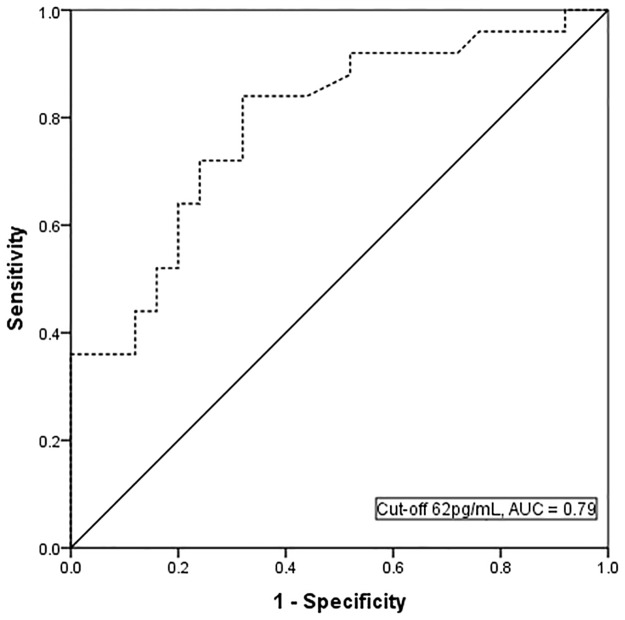
Receiver operative curve (ROC) analysis using (1→3)-β-D glucan at cut-off level 62 pg/mL to predict those with detectable endotoxin signals using LAL(-).

Apparent endotoxin signals detected using LAL(-) was higher in patients with BG levels >80pg/mL (positive test) compared to those with BG levels <60pg/mL (negative test) [0.034 vs. 0EU/mL; p = 0.004](Fig 2). Endotoxin signals in patients with BG levels >80pg/mL also tended to be higher than those with BG levels between 60-80pg/mL (indeterminate result) although this did not reach statistical significance [0.034 vs. 0.028 EU/mL; p = 0.09]. Elevated BG (>60pg/mL) were found in 58% of patients and BG levels was significantly higher in those with detectable endotoxemia compared to those with undetectable endotoxemia (78 vs. 54pg/mL, p<0.001[Table 2]). These findings lend support to the notion that the ‘endotoxin’ signals detected using LAL(-) are false positives due to interference from BG. ROC analyses revealed a strong relationship between high BG levels and apparent endotoxemia using LAL(-) (AUC = 0.79 [0.66, 0.92]; p<0.001) [Fig 3]. The optimum cut-off levels for identifying patients with apparent detectable endotoxemia were between 60-70pg/mL. BG levels >62pg/mL identified apparent endotoxemia with sensitivity of 72% and specificity of 76%. Level of agreement between patients with apparent detectable endotoxemia and those with elevated BG levels (>62pg/mL) was moderate (kappa = 0.48, p = 0.001).

### Effect of BG-blocking buffers on endotoxin spike recovery

**Fig 4:**
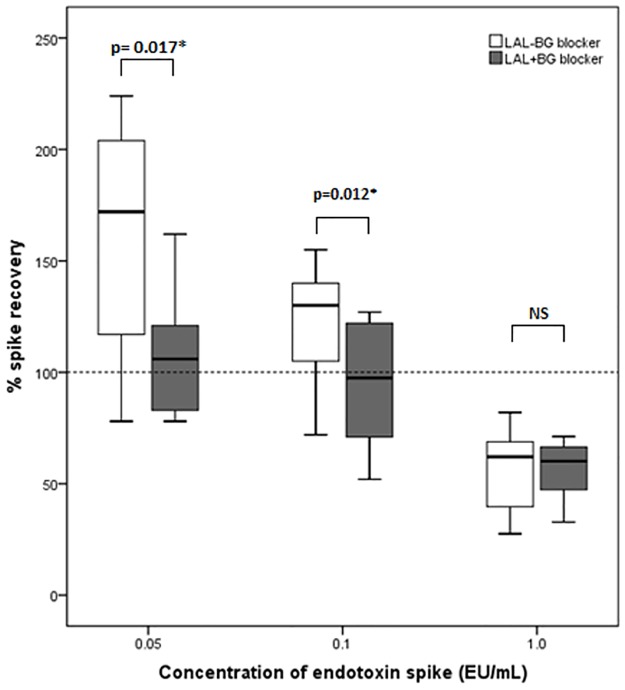
Comparison of median spike recovery between LAL(-) and LAL(+) in plasma samples spiked with endotoxin (box plots represent median values with interquartile range, hashed horizontal line denotes the expected optimum spike recovery). Spike recoveries of 50–200% are considered acceptable [31][LAL(-), standard LAL with no glucan blocking buffer; LAL(+), LAL reconstituted with Charles River^®^ ES-buffer containing 1mg/mL carboxymethylated curdlan].

Endotoxin spike recovery for samples spiked with 0.05EU/mL and 0.1EU/ml was elevated at 172% and 130% respectively using LAL(-), suggesting enhancement of the LAL assay. However, this enhancement was abolished on repeat measurement with BG-blocking buffers [LAL(+)], with spike recoveries falling closer to the expected value of 100% (106% and 97.5% for samples spiked with 0.05EU/mL and 0.1EU/ml respectively; p<0.05)[Fig 4]. For samples spiked with high concentrations of endotoxin (1EU/mL), spike recovery was broadly reduced for both LAL(-) and LAL(+), with no significant differences in spike recovery. Despite this, all spike recoveries were within industry-accepted limits of 50–200%[31] [Table 3].

**Table 3:** Spike recovery from plasma samples with undetectable endotoxin using different concentrations of glucan blocking buffers. Median spike recovery with interquartile ranges presented. Spike recoveries of 50–200% are considered acceptable [31]. Significant P values (*) indicates spike recovery significantly different from expected % spike recovery.

Concentration of carboxymethylated curdlan (mg/mL)		0	1	0.5	0.1	0.01
		Standard LAL (no glucan blocking buffer)	(Manufacturer recommended dose)			
**Concentration of endotoxin spike**	**0.05**					
**(Expected endotoxin content) [EU/mL]**						
Spike recovery (%)		172 [108.5–210]	106 [80.5–122.5]	149 [97.5–206]	220 [134.5–343.5]	848 [271–1798]
**P**		0.028*	**0.574**	0.036*	0.012*	0.012*
**Concentration of endotoxin spike**	**0.1**					
**(Expected endotoxin content) [EU/mL]**						
Spike recovery (%)		130 [102.5–142.5]	97.5 [69–124]	120.5 [73.8–202.3]	160.5 [105.5–274.3]	665.5 [170.3–1391]
**P**		**0.063**	**0.674**	**0.362**	0.036*	0.012*
**Concentration of endotoxin spike**	**1**					
**(Expected endotoxin content) [EU/mL]**						
Spike recovery (%)		62.1 [37.6–69.5]	60.1 [46.8–67]	64.9 [44.4–85.8]	67.8 [46.2–89]	99.7 [57.8–190.5]
**P**		0.012*	0.012*	0.036*	**0.05**	**0.575**

**Fig 5:**
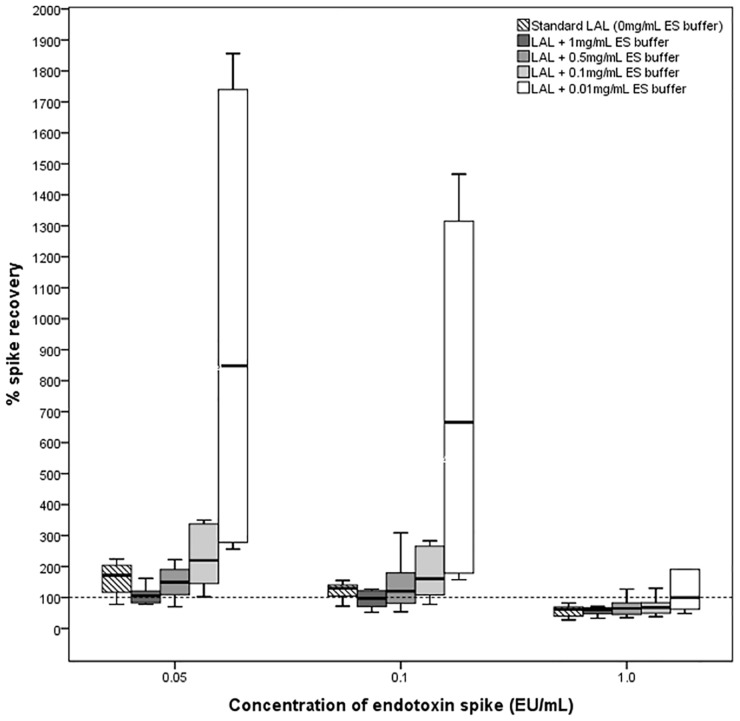
Comparison of median spike recovery between LAL and LAL reconstituted with different doses of BG blocking buffer (ES buffer). Spike recoveries of 50–200% are considered acceptable [31] [box plots represent median spike recovery with interquartile range, hashed horizontal line denotes the expected optimum spike recovery].

Assay measurements using LAL re-constituted with increasingly dilute amounts of carboxymethylated curdlan resulted in progressive enhancement of spike recovery (Table 3, Fig 5). Lower doses of BG-blocking buffer (0.01–0.1 mg/mL ES buffer) caused activation of the LAL reagent almost immediately upon reconstitution resulting in wide variability of spike recoveries obtained using lower doses of BG-blocking buffer.

### Measurement of BG in dialysis fluid

High levels of BG were observed in a significant proportion of patients and since BG leaching from cellulose-based HD membranes have been reported[32], we examined dialysers at our unit for BG contamination. Pre- and post-dialyser saline washout from the blood compartment of dialysers revealed very low level contamination. Post-dialyser washout tested positive for BG in 6/12 dialysers (range 8-22pg/mL). Median BG levels in post-dialyser washout were 4.8 pg/mL (Table 4).

**Table 4:** BG measurements of saline washout pre- and post-dialyzer.

	Pre-dialyzer (n = 12)	Post-dialyzer (n = 12)	P
(1–3)-β-D-glucan (pg/mL)	Below detection limit	4.8 [IQR 0–9.8]	0.046*

Limit of detection was 7.8pg/mL, values <7.8pg/ml was designated as 0

*denotes statistical significance P<0.05

### Clinical correlates of (1–3)-β-D-glucan

Weight and residual urea clearance correlated negatively with plasma BG levels (r = -0.33 and -0.31 respectively; p<0.05). Plasma BG correlated positively with endotoxin signals detected using LAL[–] (r = 0.55, p<0.01) but did not correlate significantly with markers of inflammation including TNF-α, IL-6, albumin or CRP. There was no relationship between BG and age, co-morbidity, dialysis vintage, dialysis adequacy as determined by Kt/V, convective volume or dialyser surface area (Table 5).

**Table 5:** Clinical correlates of (1–3)-β-D glucan, endotoxin signals detected using LAL[–] and markers of inflammation.

	Variable	1	2	3	4	5	6	7	8	9	10	11	12	13
**1**	BG													
**2**	Endotoxin LAL[–]	0.55**												
**3**	IL-6	-0.11	0.16											
**4**	TNF-α	-0.01	0.01	0.17										
**5**	CRP	-0.16	0.18	0.53**	0.05									
**6**	Albumin	-0.03	-0.14	-0.2	-0.33*	-0.08								
**7**	Age	0.09	0.14	0.2	0.01	-0.09	-0.32*							
**8**	Weight	-0.33*	-0.37**	0.21	0.02	0.21	0.05	-0.15						
**9**	KRU	-0.31*	-0.11	0.06	-0.13	0.13	0.13	0	0.47**					
**10**	CCI	-0.12	0	0.30*	0.19	0.01	-0.47**	0.76**	-0.08	0.01				
**11**	Dialysis vintage	0.14	0.17	0.01	0.32*	-0.03	-0.21	0.15	-0.3*	-0.54**	0.28			
**12**	Kt/V	0.08	0.2	-0.07	-0.16	-0.07	-0.03	0.33*	-0.15	0.33*	0.23	-0.21		
**13**	Convective volume	0.02	-0.22	-0.16	-0.16	0.04	0.15	-0.28	0.26	-0.23	-0.32*	0.12	-0.21	
**14**	Dialyser surface area	-0.2	-0.24	0.28	-0.01	0.35*	-0.11	-0.07	0.73**	0.19	0.01	-0.06	-0.15	0.27

*p<0.05

**p<0.01

BG, (1–3)-β-D glucan; IL-6, interleukin-6; TNF-α, tumor necrosis factor-α; CRP, median C-reactive protein over the previous 3 months; KRU, residual urea clearance; CCI, Charlson Co-morbidity index; Kt/V, combined dialyser and renal urea clearance normalised to body volume; Endotoxin LAL[–], Endotoxin signals detected using LAL assay without BG-blocking buffers

## Discussion

The levels of endotoxemia reported in the dialysis population are high and often appear close to or to exceed the pyrogenic threshold of 4.1–5 EU/kg in humans [13], [14]. Contrary to the high blood endotoxin levels reported in the literature, the apparent level of endotoxemia detected using LAL(-) in our population was low with a median level of 0.038–0.041 EU/mL[21]. These low levels do not appear to be due to sub-optimal pre-treatment of plasma to remove inhibitory plasma components since spike recovery from all samples was sufficient and well within the 50–200% limit (Fig 4). The plasma dilution and heat treatment conditions used in this study was similar to those used by many other authors [33].

It is possible that the high endotoxin levels reported in the literature are due to pre-analytical factors such as contamination of phlebotomy and laboratory apparatus. Most blood collection tubes are sterile but not certified to be endotoxin-free, additives in blood collection tubes may also be a source of contamination [9]. We paid meticulous attention to these factors by testing phlebotomy apparatus for endotoxin contamination and interfering factors. Only manufacturer-certified endotoxin free laboratory apparatus was used for the analysis.

False positive activation of the LAL from BG may also be partly responsible for the apparently high ‘endotoxin’ levels reported in dialysis patients. The apparent endotoxin signals detected in 50% of our cohort using LAL(-) became undetectable when the assay was repeated with LAL(+) to block factor G activation. Furthermore, endotoxin signals detected using LAL(-) were significantly higher in those with the highest blood BG levels. These finding suggest that endotoxin signals in this study detected using standard LAL are likely to be false positives due to interference from BG.

The factor G pathway of LAL was inhibited using highly concentrated carboxymethylated curdlan[25], [34]–itself a BG. The interaction between BG and factor G is complex[35]. Lower molecular weight BG structures such as laminarin and curdlan degradation products have factor G activating properties at low concentrations, but become inhibitory at high concentrations. Consistent with this, we observed an increase in endotoxin spike recovery as the concentration of the BG-blocking buffer was progressively reduced and very dilute concentrations of BG-blocking buffer caused coagulation of the LAL reagent almost immediately upon reconstitution. This peculiar phenomenon is the basis of commercial BG-blocking agents used to eliminate BG interference in LAL endotoxin detection[17] and BG-blocking agents typically consist of solutions containing highly concentrated BG.

Highly concentrated carboxymethylated curdlan used as a BG-blocking buffer does not appear to affect the ability of LAL to detect endotoxin[25], although previous studies have tested this in plasma samples spiked with relatively high concentrations of LPS. Since endotoxin content in our patients with detectable endotoxemia was relatively low (median 0.038EU/ml), to ensure that the BG-blocking buffer used in this study did not interfere with true endotoxin detection at this level of endotoxemia, we examined the effect of the BG-blocking buffer on the LAL assay’s ability to recover endotoxin from plasma spiked with lower amounts of endotoxin (0.05–0.1EU/mL). Spike recovery was enhanced (130–172%) using LAL(-), however on repeat measurement using LAL(+), this enhancement was abolished with spike recoveries falling closer to expected values (97.5–106%). This suggests that within this range of endotoxemia, there could be enhancement of the LAL assay by BG present in the blood, supporting the hypothesis that ‘endotoxemia’ detected in HD patients may be artefactual due to BG interference. This also demonstrates that BG-blocking buffers used in this study do not interfere with the sensitivity of the LAL assay to detect endotoxin since spike recovery using LAL(+) was close to 100%. All spike recoveries obtained were within the industry-specified limits of 50–200%. As the LAL test is a biological assay and is subject to inherent variability, wide-ranging spike recoveries are permitted[31], therefore this may lead to LAL users accepting results of endotoxin measurement and not taking steps to rule out BG positive interference.

In samples spiked with a high concentration of endotoxin (1EU/mL), spike recovery was broadly reduced and there was no significant difference in spike recovery between LAL(-) and LAL(+), possibly reflecting that the contribution of residual BG signal was only a small proportion of the overall signal when higher concentration of LPS is used. Overall spike recovery was broadly reduced compared to samples spiked with smaller amounts of LPS, We are unable to explain this observation although improvement of endotoxin recovery from plasma using detergent have been reported[21], [36], [37] and since detergents alter the aggregative state of endotoxin in high protein content solutions[38], this suggests that the structure and potency of endotoxin molecules may be altered when a large amount of endotoxin is spiked directly in plasma.

Elevated levels of blood BG were found in a large proportion of patients. The clinical significance of high blood BG levels in-vivo is unclear. There was no relationship between BG with markers of inflammation in this study although only two pro-inflammatory cytokines were studied. A broader inflammatory profile may have been useful as BG has been shown to stimulate release of a number of other pro-inflammatory cytokines such as IL-8 [39], [40]. Different cut-off levels of BG are used as a diagnostic marker in invasive fungal infections and in-vitro evidence suggests that BG may potentiate enhanced Toll-like receptor-induced cytokine production[41]. However, the use of BG as adjunctive therapies for treatment of malignancies has been reported [17], [18], [42], [43]. The source of high BG in our study is unknown. Though we found no evidence in single-pass experiments that the dialysis membranes were involved in significant BG generation, we cannot discount the possibility that cumulative treatments over many years may increase BG levels, however there was no correlation between BG levels and dialysis vintage. The gut is a potential source of high BG levels given reports of translocation of intestinal luminal contents due to disturbed gut permeability in uraemia[44–46], although we did not observe a relationship between BG levels and history of gastrointestinal disease. We found an inverse relationship between BG with weight and residual kidney function. Animal studies suggest that BG are cleared mainly by the liver[47], [48] and degraded oxidatively in the reticuloendothelial system[17], [49], with minimal excretion by the kidney. The role of kidney function in BG excretion is poorly understood[47] and requires further investigation.

The strengths of this study include the use of a kinetic turbidimetric LAL assay which we found to have good performance for endotoxin detection in uraemia[21], [22]. Meticulous precautions were taken to minimise risk of environmental contamination of plasma samples from phlebotomy and laboratory apparatus. Plasma samples were examined for BG using a detection assay which has been extensively validated as a diagnostic adjunct of invasive fungal infections and cleared for marketing by the FDA[26]. Due to the inherent variable nature of LAL testing [30], measures were taken to minimize inter-batch and inter-plate variation by conducting the analysis using the same batch of LAL and conducting assay measurements on the same microplate for each subject. The limitations include the relatively small sample size and the use of reagents from only one manufacturer. It is unclear whether a similar conclusion would be reached if LAL reagents and BG-blocking buffers from other manufacturers were used. However, a previous study similarly found a low prevalence of detectable endotoxemia in HD patients when measurements were carried out using LAL devoid of factor G[19]. Finally, endotoxin used for spiking experiments should ideally have been conducted using natural endotoxin rather than control standard endotoxin (LPS) for assessment of spike recovery, however natural endotoxin is difficult to standardise which precluded its use in this study.

In conclusion, BG presence represents a significant limitation with the LAL assay for endotoxin detection in dialysis patients. Our data demonstrate that endotoxin measurement, in a setting free of BG contribution from dialyzer equipment or dialysis fluids, suffers from significant, positive BG interference, especially at very low levels of reported endotoxin. Accordingly, endotoxin measurements in dialysis patients should be carried out with LAL rendered insensitive to BG. Raised BG levels were found in a significant proportion of HD patients. The source and clinical significance of this is unknown and warrants additional study.

## Abbreviations

HD: Hemodialysis
LAL: Limulus Amebocyte Lysate
BG: (1→3)-β-D glucan
TNF-α: Tumor necrosis factor-α
IL-6: Interleukin-6
CRP: C-reactive protein
HIV: Human immunodeficiency virus
LAL(-): standard Limulus Amebocyte Lysate reconstituted without (1→3)-β-D glucan blocking buffer
LAL(+): Limulus Amebocyte Lysate reconstituted with (1→3)-β-D glucan blocking buffer
LPS: Lipopolysaccharide (control standard endotoxin)
CV: Coefficient of variation
